# A Mathematical Model to Capture Complex Microstructure Orientation on Insect Wings

**DOI:** 10.1371/journal.pone.0138282

**Published:** 2015-10-07

**Authors:** Delyle T. Polet, Morris R. Flynn, Felix A. H. Sperling

**Affiliations:** 1 Department of Biological Sciences, University of Calgary, Calgary, AB, Canada; 2 Department of Biological Sciences, University of Alberta, Edmonton, AB, Canada; 3 Department of Mechanical Engineering, University of Alberta, Edmonton, AB, Canada; 4 Applied Mathematics Institute, University of Alberta, Edmonton, AB, Canada; Coastal Carolina University, UNITED STATES

## Abstract

Microstructures on insect wings can promote directional drop shedding, and the local orientation of these structures is expected to facilitate drop removal. However, microstructures may exhibit very different orientations at different locations on the wing. Using the march fly *Penthetria heteroptera*, we propose that local orientation of small hairs (microtrichia) reflects a balance of three nonexclusive strategies: (1) preventing water from becoming stuck in intervenous grooves (microtrichia point upslope), (2) shedding water off the wing as readily as possible (microtrichia point towards the nearest edge), and, (3) shedding water away from the body (microtrichia point distally). We present evidence for all three and show that local microtrichial orientation is seldom determined by any one factor. We develop a mathematical model that employs factor-specific weighting values determined *via* optimization. Our predictions are tested against the orientation of microtrichia randomly sampled from a *P. heteroptera* specimen. Using the best-fit weighting parameters, the model displays a median residual of 20°; no residual is greater than 46°. The model also reproduces qualitative aspects of microtrichial orientation, such as bifurcation midway between veins and convergence toward peaks. This strong correspondence between modelled and observed orientation supports the role of microtrichia as directional antiwetting devices and highlights the importance of considering both function and wing geometry to explain the organization of natural microstructure arrays.

## Introduction

Many insects display dense arrays of micro or nanostructures that promote antiwetting on wing surfaces. These structures can take on a variety of forms, such as bumps, asters, scales and hairs [1–4]. Hairs and other micro and nanostructures on insect cuticle have attracted much attention recently, due to their application to bioinspired superhydrophobic surfaces [5, 6]. For example, small structures can turn a chemically hydrophobic surface into a superhydrophobic surface (contact angle greater than 150°) through the Cassie-Baxter effect, wherein pockets of air become trapped between the water interface and the insect cuticle [7].

Biological microstructures often have a nonrandom orientation, which can promote shedding of water drops and contaminants in one direction over another. Zheng *et al*. [3] showed that small scales on a *Morpho aega* butterfly wing (Hubner 1982) prevented drops pushed against the grain from detaching, but promoted drop shedding along the grain. Prakash and Bush [8] showed similar properties in the setae of a water strider leg. Directional surfaces can help an insect detach itself efficiently from aquatic surfaces or remove heavy drops from its wings and body.

While microstructures on insect wings may have a nonrandom direction locally, they often display variation in orientation across the wing surface. The developmental and genetic basis of microtrichial orientation has been explored in *Drosophila spp.* [9–12]; however, the functional adaptation of local variation is unknown- though Adler *et al*. [10] noted mutant flies with random microtrichial orientation appear to get stuck in wet substances more easily and struggle to exit their pupal cases, suggesting adaptations to reduce wetting and adhesion.

If microtrichia (and microstructures in general) form directional wetting surfaces, then we would expect variation in orientation to promote efficient shedding of water and contaminants from the wing. One strategy would be to locally orient the array towards the nearest edge, thereby promoting quick drop shedding. There are, however, two main problems with this strategy. First, shedding drops from the wing to the body would be counterproductive in terms of load reduction. Second, grooves between veins on highly corrugated surfaces could potentially trap drops, and drops may shed off the wing more easily if water were kept out of these grooves, regardless of their distance to a wing edge.

We therefore propose that local microstructure orientation on insect wings is a result of balancing three strategies that sometimes compete: (1) preventing water from becoming stuck in intervenous grooves, (2) shedding water off the wing quickly and (3) shedding water away from the body. First, we establish microtrichia as antiwetting structures in a large dipteran (*Penthetria heteroptera*, Say 1823). Next we demonstrate an association between large scale topography (*i.e.* the local slope of the wing) and local microtrichial orientation in this same species. We also demonstrate that microtrichia orient towards the nearest edge as well as distally overall, though no one factor is responsible for all the variation observed. Finally, we develop a simple mathematical model that captures these three effects and demonstrate that this model can reproduce the majority of variation of microtrichial orientation observed on the dipteran wing.

## Materials and Methods

### Collection and preservation

Adult *Penthetria heteroptera* (Diptera: family Bibionidae) were collected September 9^th^ 2012 from private land near Lloyd Creek Natural Reserve, Alberta, Canada, after obtaining permission from the land owner. The collection of specimens complied with all provincial and national regulations, and did not involve endangered or protected species. Samples were preserved in a freezer at -80°C until use. When removing wings from specimens, the experimenter was careful to seize the wing only near the wing base.

### Scanning electron microscopy

**Fig 1:**
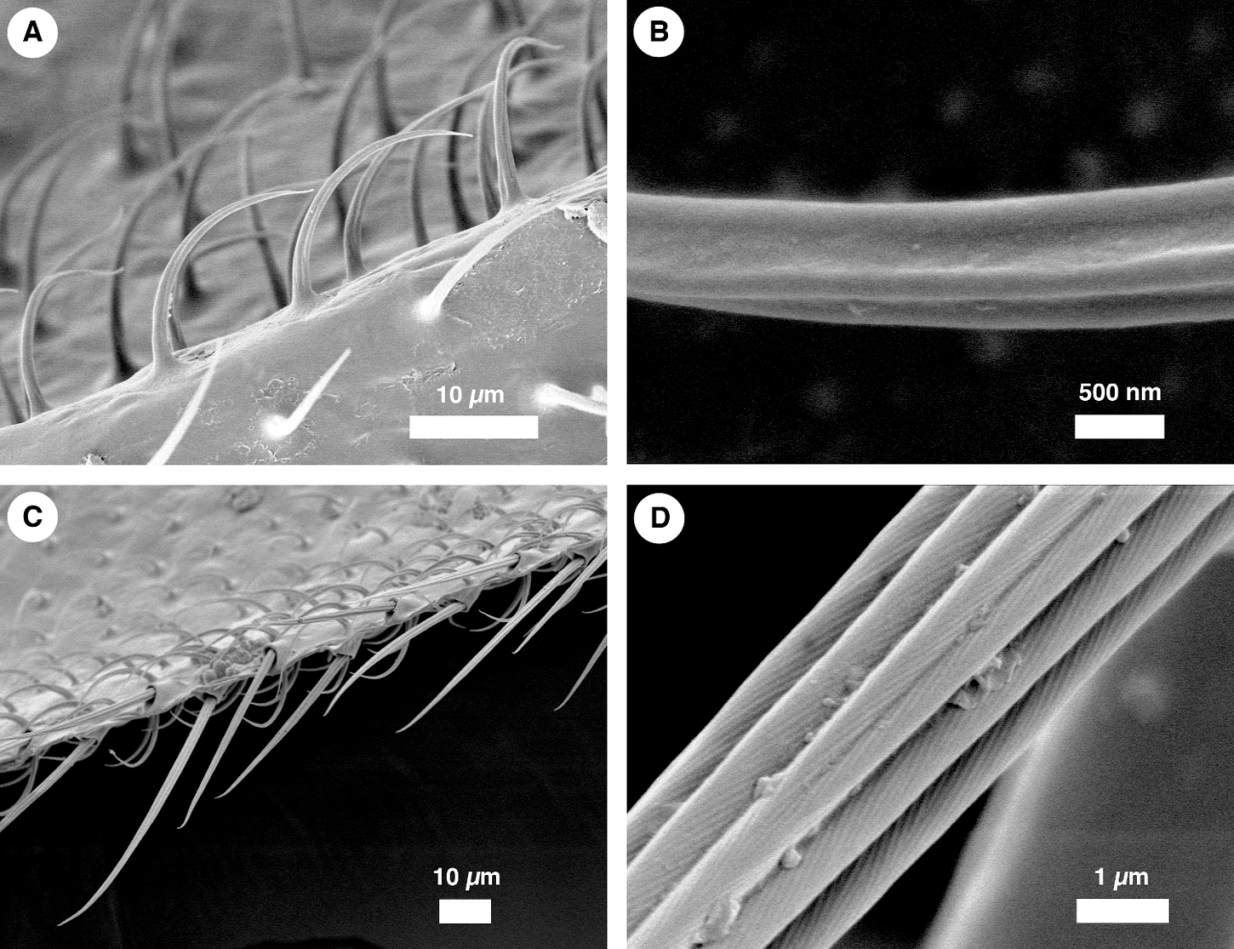
Scanning electron micrographs of hair-like structures on a *Penthetria heteroptera* wing. (A) A side view of microtrichia. These microtrichia cover nearly the entire wing surface. (B) Each microtrichium displays three parallel grooves running along the length of the hair. Such grooves are hierarchical structures, which have been shown to enhance wetting abilities [13]. (C) A side view of setae, which run along the margin of the wings. (D) Each seta displays two sublevels in the structural hierarchy: cord-like structures that run along the length of the seta, and, superimposed on these cords, small grooves running at an angle to the cord.

For scanning electron microscope (SEM) imaging, wings were gently placed, dorsal-side up, on a pin-type stub with double-sided carbon tape. Wings were gently pressed flat against the tape with a soft and flat plastic implement. The wing and stub were sputter coated with gold and imaged using a JEOL 6301F Field Emission Scanning Electron Microscope. One pair of wings from a *P. heteroptera* female was imaged (Fig 1).

### Contact angle and drop rolloff angle measurements

Double-sided tape was placed flat on a microscope slide. Wings were removed from a preserved specimen, placed dorsal-side-up on the tape, and gently flattened with a soft plastic implement. 5 *μ*L distilled water drops were produced from a 2 mL syringe with a regular bevel, 27 gauge needle by slowly applying manual pressure to the plunger until a water drop detached from the needle tip. The syringe was held securely in place by a stand. Drop contact angle on the wing was measured in ImageJ from profile view photos of static drops taken with a Canon PowerShot Elph 300HS camera with an attached Bushnel 8 × 20 mm monocular.

For drop rolloff angle measurements, 10 wings from five males were flattened on microscope slides and positioned on a Newport GON65-U goniometric stage, such that the torsional axis was oriented downslope. Distilled water drops produced using the same method as above were placed in the center of the level wings. Pictures were taken at 1° increments until the drop was observed to roll off the wing or the maximum inclination of 10° was reached. This process was repeated a minimum of four more times in both the distal and proximal tilting directions. Three wings (two right and one left) were rejected from the analysis because the cuticle was folded over near the wing centre, creating a strong pinning point.

For the remaining wings, images of drops were analyzed and the drop rolloff angle for a particular trial was taken as the lowest angle at which the drop contact line was observed to advance downslope compared to its starting position on the wing. If depinning had not occurred by the 10° mark, the rolloff angle was set at 11°. Drop rolloff angles in the distal and proximal directions respectively were taken as a weighted average of mean rolloff angle per wing in a given direction. The weights were chosen as the reciprocal of the standard error of the mean rolloff angle in a particular direction for a given wing. The ratio of force of retention (*R*_*FR*_) in the proximal *vs*. distal directions was computed from

$$R_{FR}=\frac{\sin\beta_{p}}{\sin\beta_{d}},$$ (1)

where *β*_*p*_ and *β*_*d*_ are the rolloff angles in the proximal and distal directions, respectively.

### Light microscope imaging and microtrichial orientation sampling

Wings flattened on microscope slides were imaged with a Nikon Coolpix 8400 camera attached to an Olympus SZX16 microscope at 10× magnification. This same setup was used to image wing sections (described below). The experimenter was careful to keep the microtrichia of the upper surface in focus, and the underside microtrichia out of focus. Images from the same wing were stitched together in Photoshop (v. CS2) to form a complete compound image of each wing (see S1 Fig).

**Fig 2:**
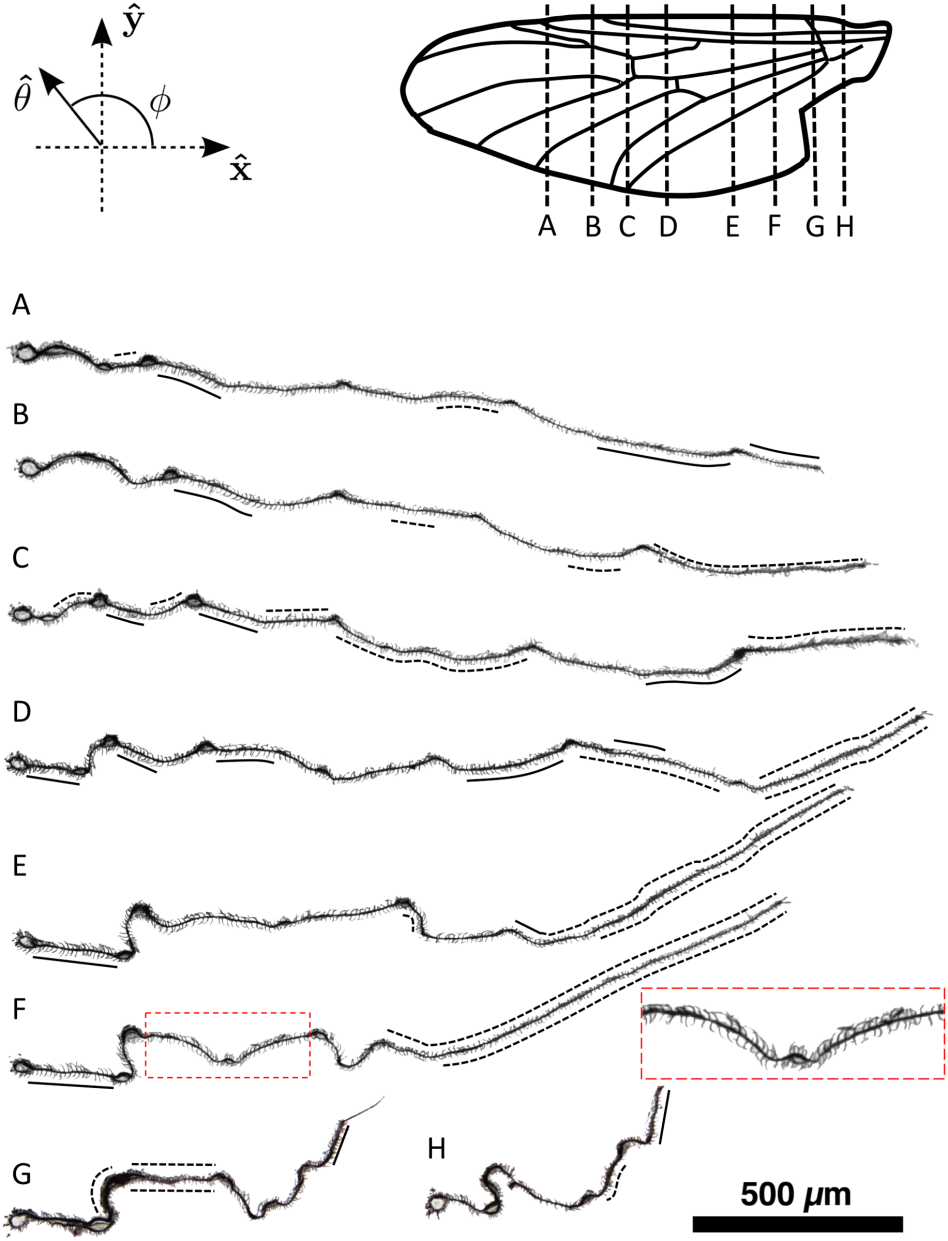
Microtrichia point predominantly upslope in sections of a male *P. heteroptera* wing. (*Top*) Venation of a male *P. heteroptera* wing, based on Hardy [15], with dashed lines indicating the approximate location of cross-sections A-H shown below. The $\hat{x}$ direction points proximally along the torsional axis. The wing lies in a plane formed by $\hat{x}$ and $\hat{y}$. Microtrichial orientation is denoted by the vector $\hat{\theta }$, whose angle relative to the $\hat{x}$ axis is given by *ϕ*. (A-H) The *P. heteroptera* wing forms a rugged surface characterized by peaks and valleys. In all sections, hairs point overall towards the nearest peak on the dorsal surface, or towards the peaks on the ventral surface if the wing were flipped upside down. The inset on the right of section F zooms in on the red rectangle to highlight this phenomenon, and shows upslope orientation on the dorsal surface away from the medial vein and reverse orientation on the ventral surface. The areas that are exceptions to the uphill-orientation bias are highlighted with solid black lines; ambiguous areas are highlighted with dashed black lines. The scale bar refers to cross-sections A-H.

The orientations of microtrichia were assessed relative to the torsional axis of the wing (see Fig 2). The torsional axis was determined on a male wing using the method described by Norberg [14].

A composite image of the upper wing surface was rotated so that the image horizontal axis was parallel to the torsional axis. 24 quadrats 75 × 75 px (56 × 56 *μ*m) were centred at random coordinates on the wing determined by a pseudo-random number generator. Quadrat size was chosen so as to minimize quadrat-edge effects while easing data collection. The orientation of each microtrichium was determined from the orientation of the line connecting the centre of the hair base to the hair tip, and was measured relative to the horizontal axis of the image in ImageJ. If the centre of a hair base lay within the quadrat, that microtrichium was included in the quadrat sample; otherwise it was excluded. Hairs on veins were not sampled. The local orientation at the location of each quadrat centre was taken as the arithmetic mean orientation of all hairs within the quadrat.

### Sectioning

Wing samples were fixed with formalin and placed in melted wax. Once cooled and solidified, the wax was trimmed and reoriented so that chordwise sections could be taken. Target locations for collecting sections for imaging were determined beforehand (Fig 2). Ten 14 *μ*m sections were taken near these targets.

At each target location, the section showing the clearest detail was chosen for imaging. Images of sections were stitched together using pairwise stitching in ImageJ [16]. Images were rotated so that the upper wax edge of the section was aligned with the horizontal. The central section (section D in Fig 2) was further rotated so that it appeared horizontal. All other sections were rotated by the same amount.

Wax surrounding each section was digitally removed from images using the magic wand feature in Photoshop (v. CS2 for Macintosh). Any obvious wax portions that remained were removed manually in Photoshop. If there was any ambiguity as to whether an object was wax or cuticle, that feature was untouched. The experimenter was careful to ensure that no wing features, including microtrichia and setae, were excluded by this procedure. Using the sections as a guide, wing veins were assessed as local topographic maxima or minima.

### Data processing

Statistical analyses on sampled microtrichial orientations were performed using custom MATLAB scripts (v. 2014b for Macintosh). Model optimization was also performed in MATLAB to determine the best-fit values of *W*_1_ and *W*_2_. *W*_1_ and *W*_2_ represent weighting factors for an upslope microtrichial orientation and an orientation to the nearest-edge, respectively; these parameters are discussed in depth in the “Modelling” section below. For optimization, values of 0 ≤ *W*_1_ ≤ 2 and 0 *μ*m ≤ *W*_2_ ≤ 1493 *μ*m were tested, with resolutions of 0.001 in *W*_1_ and 0.75 *μ*m in *W*_2_. The modelled microtrichial orientation at the location of each quadrat centre was compared against the observed average orientation of the corresponding quadrat, and the sum-square-residual (SSR) between modelled and observed orientation was computed for each combination of *W*_1_ and *W*_2_. The optimal combination of *W*_1_ and *W*_2_ was taken as the pair of values that minimized the SSR relative to observations. For the optimization procedure, as well as statistical analyses involving nearest-edge orientation, a single quadrat whose centre lay outside the wing was not used, as the model would then be searching for orientations of microtrichia outside the wing surface. Data is available as supplemental files using a comma-delimited format (S1–S4 Files).

## Results and Discussion

### Scanning Electron Microscopy and contact angle measurements

Fig 1 shows scanning electron micrographs from a wing of a *P. heteroptera* female. Microtrichia and setae (Fig 1A–1B and 1C–1D, respectively) were the only two hair types on the wing. Both types contain hierarchical substructures. On microtrichia, these were smooth, 300 nm wide grooves that ran along the length of the hair (Fig 1B). Setae exhibited two levels of subhierarchy: large cords running along each seta and grooves running diagonal to the cords (Fig 1D). Hierarchical structures that serve antiwetting purposes are widespread among insects [4, 13, 17], and likely serve the same function here.

Microtrichia cover nearly the entire surface of the wing, except for part of the subcosta and claval furrow. In contrast, setae are distributed only along the wing margin and along the radial vein (except R2+3). Therefore, microtrichia would be the primary antiwetting structure on the insect wing surface.

To establish the role of microtrichia as antiwetting structures, measurements of static drop contact angles were performed on flat portions of the wing. Contact angles were measured to be 150±2° (mean ± standard error). Such a high contact angle cannot be produced by purely chemical means on insect cuticle, and is only realizable by virtue of cuticular microstructures [17], supporting the antiwetting function of microtrichia.

To characterize whether the surface displayed directional adhesion, drop rolloff angle was compared in the distal *vs*. proximal directions, and was found to be 5.8 ± 0.5° and 8.5 ± 0.4° respectively (weighted mean ± standard deviation of the weighted mean; cumulative sample size = 40 and 39, respectively). The ratio in the force of retention in the proximal *vs*. distal directions (Eq 1) is thus 1.47, indicating a strong bias favouring drop shedding in the distal direction. This directional property helps drops avoid shedding off the wing proximally and reattaching to the body.

### Sectioning and quadrat sampling of microtrichial orientation

To establish the relationship between microtrichial orientation and wing topography, chordwise wing sections were examined (Fig 2). Maximum corrugation relative to maximum chord length was 6%, close to the proportions found by Rees [18] of 8% in *Tipula oleracea* (Linnaeus 1758), and 7% in *Episyrphus balteatus* (De Geer 1776). Hairs viewed on the sections in Fig 2 orient upslope in general. The inset next to section F in Fig 2 shows a close up of this section to clarify the pattern of microtrichial orientation. Here the medial vein is a local minimum, bounded by peaks on either side. On the dorsal surface, microtrichia point away from the medial vein and toward the adjacent peak. Because the medial vein would represent a peak if the wing were flipped upside-down, microtrichia orient towards the medial vein on the ventral surface.

**Fig 3:**
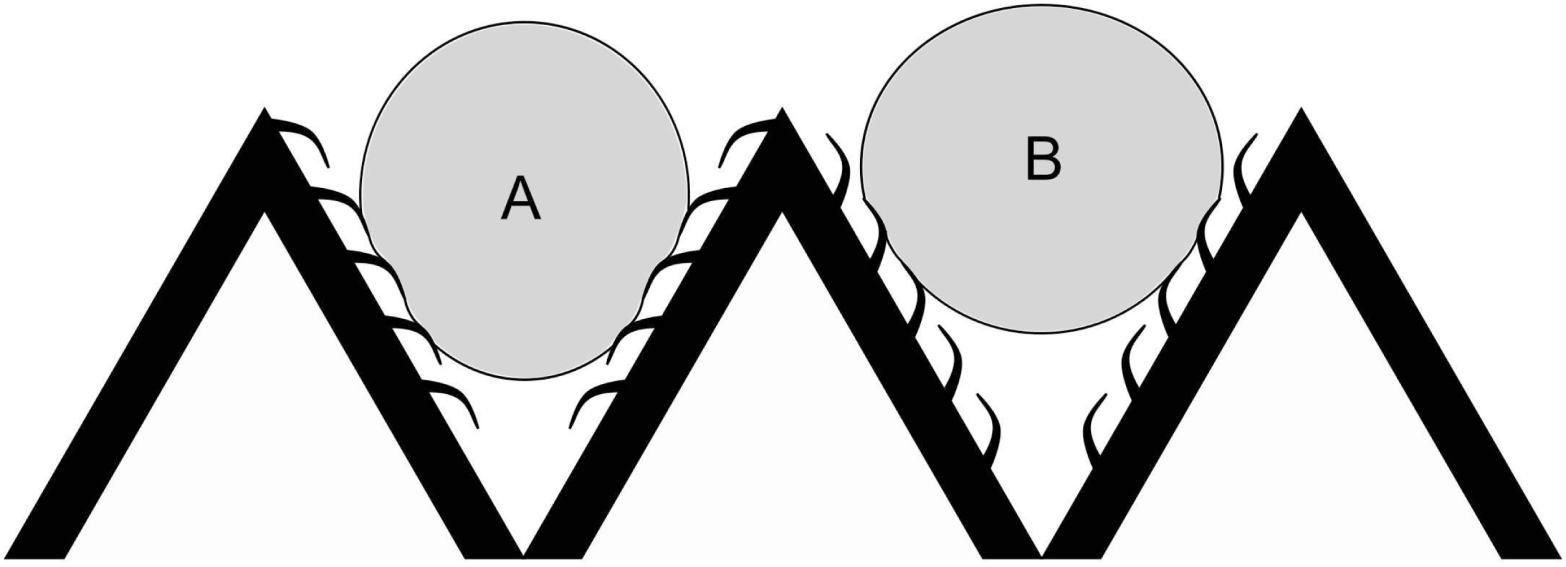
The functional rationale of upslope orientation. A schematic rationalizing the function of upslope oriented hairs in keeping water raised above the surface of a corrugated wing. (**A**) A downslope orientation would allow deeper penetration of drops into grooves. With the interface pinning preferentially against the grain of the hairs, the drop would be difficult to remove in this configuration. (**B**) An upslope orientation reduces penetration of water drop into grooves between local maxima, easing the release of these drops from the wing.

Fig 3 shows the functional rationale behind the upslope bias. Microtrichia oriented downslope would allow the air-water interface to descend deeper into the groove formed between peaks on the wing surface, leading to greater cuticle-water contact and thus greater adhesion (Fig 3A). If microtrichia are instead oriented upslope, water is prevented from descending too far into the groove, reducing adhesion and promoting water shedding (Fig 3B). Thus upslope-oriented microtrichia could have a functional role in reducing water adhesion on the insect wing.

This upslope trend was not observed everywhere. Any portion of the sections in Fig 2 where upslope-oriented hairs were not observed or where the orientation was ambiguous are highlighted with solid and dotted black lines, respectively. Overall, the degree of highlighting is relatively modest and is almost absent in come sections, *e.g.* A, B, and H. The orientation of microtrichia in the anal region of the wing (right-most portion of sections D, E, and F) is ambiguous, suggesting that this area does not have a preferred upslope orientation. Because this region is more flexible than others, there is no static depression in which drops may become stuck; thus, an upslope orientation may be less important here. However, because the sections of Fig 2 are not random samples of the wing, statistical analyses could not be performed to exclude the possibility that the observed upslope bias could arise from random orientation. Rather, statistical analyses were performed on random quadrat sampling, as described below.

Based on the sections in Fig 2, local maxima on the wing (*ridge peaks*) were identified. The direction from the centre of each quadrat to the nearest point along a ridge peak was used as a proxy for the local upslope direction. In 16 of 24 quadrats, the microtrichia pointed more upslope than downslope on average. Under a binomial distribution, there is only an 8% chance that this or an even stronger upslope bias would be observed from randomly oriented microtrichia in a random sample of 24 quadrats. Thus it is most likely that the overall upslope bias is a real effect. Because not every hair pointed upslope, however, additional factors are at play in determining the local microstructure orientation.

Hairs close to the wing margin (or edge) were observed to orient normal to the margin; another factor that appears important in determining local orientation. In 18 of 23 quadrats, the microtrichia pointed, on average, more toward the nearest edge than away from it. There is only a 0.5% chance that, in a random sample of 23 quadrats, an equal or greater proportion displaying edge orientation would be observed if microtrichia were randomly oriented. Thus, the bias towards the nearest edge appears to be another real effect. As quadrat distance to the nearest edge increases, so too does the residual between the angle of orientation to the nearest edge and the angle of microtrichial orientation (*r* = 0.52 and *p* = 0.01 from a least-squares linear fit). However, the least-squares linear fit exhibits *R* ^2^ = 0.27, indicating that distance to the nearest edge explains only a small portion of the observed variation in microtrichial orientation.

A distal bias in microtrichial orientation has been noted in *D. melanogaster* (Meigen 1830) [11] and can be observed in many dipteran species (personal observation). Zheng *et al*. [3] also noted that scales oriented “radially outward” from the body in *M. aega* and that drops roll most easily away from the body, suggesting that a distal orientation of microstructures may reduce the likelihood of drop shedding onto the body. Of the 462 microtrichia sampled on the *P. heteroptera* wing, 461 pointed more distally than proximally. This indicates a very strong bias for microtrichia to point away from the body, preventing drops from shedding onto the body from the wing. The only hair that oriented proximally exhibited *ϕ* = −87.5°; a very small proximal bias. In this case, the hair was close to a ridge peak, and the upslope vector pointed proximally.

The *P. heteroptera* wing displays 47% greater drop adhesion in the proximal compared to distal direction. Greater adhesion in the proximal direction is expected given the observed distal bias, and supports the role of microtrichia as directional antiwetting devices. However, it does not definitively exclude potential contributions by other features (*e.g.* the greater density of veins near the base).

The analysis of sections and sampled quadrats shows strong upslope, nearest-edge, and distal biases; however, few hairs pointed precisely upslope, precisely to the nearest edge or precisely distally. We seek a simple mathematical description of how these factors counterbalance in determining the local orientation observed in each quadrat, which could be used to predict local microtrichial orientation anywhere on the wing, and could even be extended to other species. This model is established in the next section.

### Modelling

Our mathematical model denotes hair direction as a unit vector in two dimensions ($\hat{\theta }$; see Fig 2– note that we use bold variables to designate vectors and hats to designate unit vectors). The model takes into account three strategies to remove water from the wing, each weighted by a constant scaling factor. The first strategy is to prevent water from settling in the grooves between peaks by biasing the orientation upslope- a general trend that was observed in quadrat samples. This strategy is described by $W_{1}\hat{u}$, where $\hat{u}\left(x,y\right)$ is the local normalized upslope gradient (taken as the direction to the nearest ridge peak in the present study), and *W*_1_ is the unitless weighting factor for the upslope bias.

The second strategy is to minimize the horizontal distance drops must travel to shed off the wing. Morphologically, this strategy manifests as an orientation towards the nearest edge. In the middle of the wing, the distance to one edge relative to any other may be comparatively small, and so no one direction would take priority. Therefore, we expect this strategy to become increasingly favourable closer to an edge. We model this strategy as $\frac{W_{2}}{{\left\|D\right\|}^{2}}D$, where **D**(*x*,*y*) is the displacement vector to the nearest edge (with units of *μ*m) and *W*_2_ is the weighting factor for the edge-weighting term (in μm). Note that this term is unbounded as ‖**D**‖ → 0 *μ*m, meaning that microtrichia on the margin point exclusively towards the nearest edge according to the model.

Finally, because shedding water off the wing and onto the body is disadvantageous, there is a distal bias to microtrichial orientation in *P. heteroptera*, similar to other species. We model this term as $\hat{B}=\left(-1,0\right)$, a distal unit vector. No weighting factor needs to be applied here because *W*_1_ and *W*_2_ can be set to represent the respective importance of upslope bias and edge proximity in proportion to the distal bias.

The complete model is given as

$$𝓒=W_{1}\hat{u}+\frac{W_{2}}{{\left\|D\right\|}^{2}}D+\hat{B},$$ (2)

$$\hat{\theta }=\frac{𝓒}{\left\|𝓒\right\|}.$$ (3)

**Fig 4:**
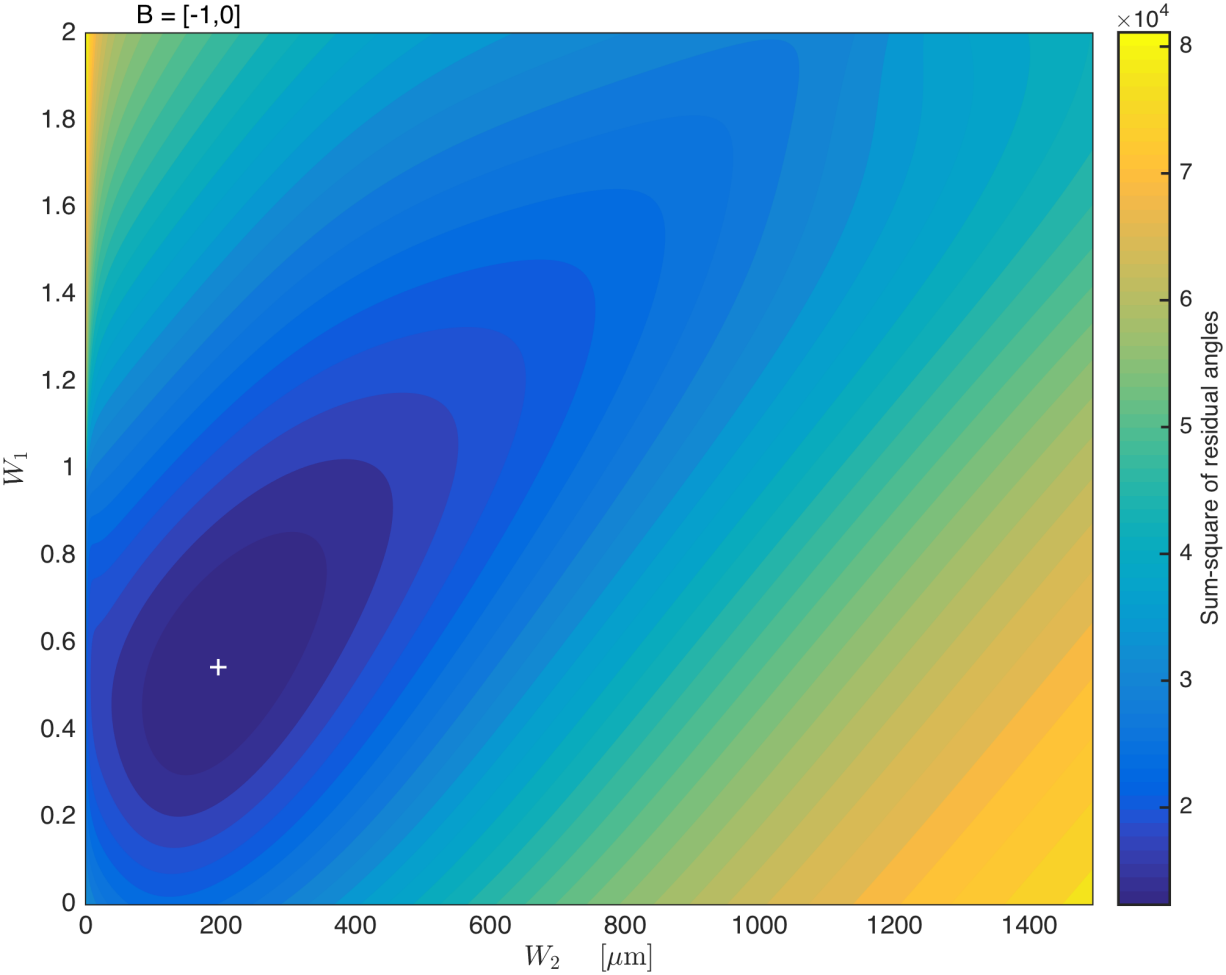
Optimization of weighting factors for the upslope bias (*W*_1_) and nearest-edge bias (*W*_2_). Colours correspond to sum-squared-residuals (SSR, in degrees squared) of the predicted *vs*. observed microtrichia orientation for particular combinations of *W*_1_ and *W*_2_. The optimization procedure converges on a single optimum in *W*_1_ and *W*_2_ (marked with a white cross) indicating the minimum SSR between predicted and observed local microtrichial orientation.

We use a brute-force optimization procedure to determine the sum-square-residual (SSR) of orientations predicted by the model through different combinations of *W*_1_ and *W*_2_ compared to observed orientations from quadrat sampling. The results are shown in Fig 4, which displays a heat map of SSRs. There is a unique optimum at *W*_1_ = 0.544 and *W*_2_ = 196 *μ*m. These optimal values for *W*_1_ and *W*_2_ are applied to the model (Eqs (2) and (3)) for the remainder of this manuscript.

**Fig 5:**
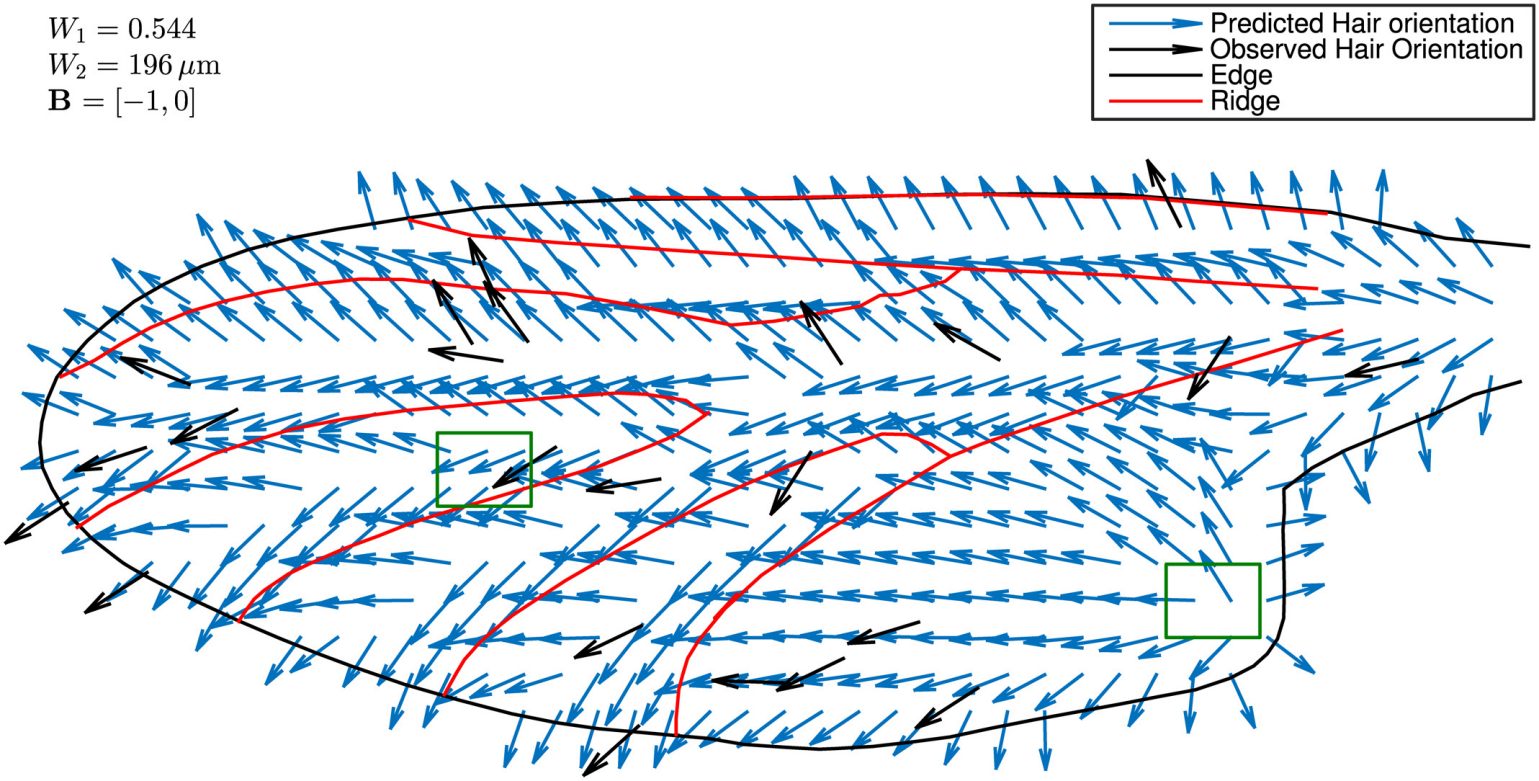
Modelled microtrichia orientation on the *P. heteroptera* wing. Modelled local microtrichial orientation (blue arrows) qualitatively match the observed microtrichial orientation (black arrows). Microtrichia orientation was measured at random locations on the wing, chosen using a random number generator. Wing edges and ridge peaks are denoted with black and red lines, respectively. The regions bounded by the left and right green rectangles are examined in detail in Fig 6, and represent zones of robust and poor model agreement, respectively. The values of the three parameters used in the model, corresponding to the best agreement with observed orientations, are given at the top-left of the figure.

**Fig 6:**
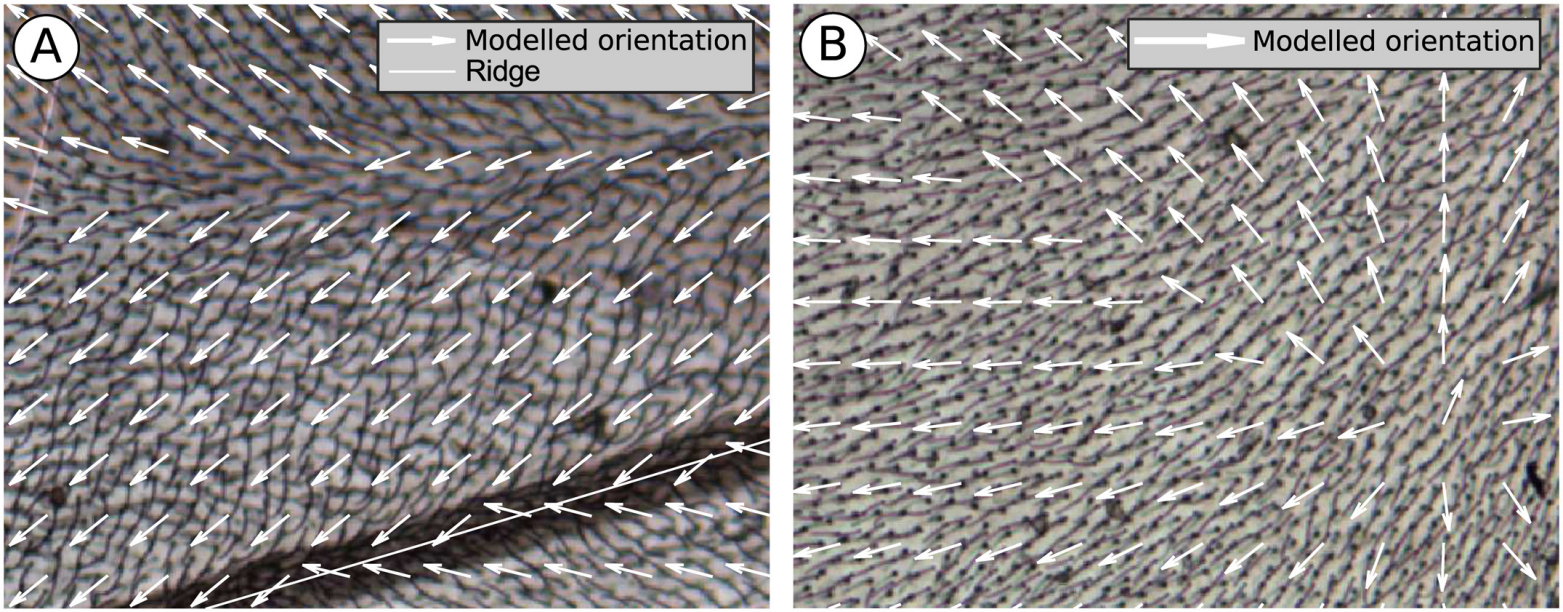
Examples of both good agreement and problem areas for modelling microtrichia orientation. (A) The model displays good qualitative agreement in most regions of the wing. In the region shown (leftmost green rectangle in Fig 5), the model correctly predicts hair divergence midway between two ridges, as well as convergence towards a ridge. Thus the model captures a high degree of local complexity with only three factors affecting orientation. (B) The model can generate artifacts near edges, when the edge-weighting term dominates the other terms in Eq (2). Shown is a portion of the anal region denoted by the rightmost green rectangle in Fig 5. The model predicts divergent orientation in this region, which was not observed. Also predicted is a bifurcating region that roughly follows a diagonal line, due to the sharp corner on the (offscreen) anal margin.

The model yields complicated variations in local microtrichial orientation (Fig 5) that could not be produced by considering $\hat{u}$, **D**, or $\hat{B}$ alone. Qualitative aspects of microtrichial orientation are captured by the model. Hair orientation was observed to bifurcate approximately midway between adjacent peaks. This feature is reproduced well by the model, as is the observed convergence towards peaks. Fig 6A is a close-up view of a distal region near a peak (leftmost green rectangle in Fig 5), where mid-groove bifurcation and convergence to the peak can be observed both in the biological specimen and in the superimposed model predictions.

However, the model also generates some artifacts that were not observed in specimens. In the anal area (in particular the rightmost green rectangle in Fig 5), the model predicts a zone of divergent microtrichia, which is absent in the microscope image of Fig 6B. This is because the edge-weighting term in Eq (2) becomes dominant close to the edges. In the anal area, the direction towards the nearest edge is posterior, and so the distal bias is overwhelmed by edge-weighting. A further complication is that the edge displays a sharp corner along the anal margin. Therefore the displacement-to-edge vector **D** displays a sharp bifurcation along a diagonal line from the corner (Fig 6B). However, no such bifurcation is observed in actuality.

**Fig 7:**
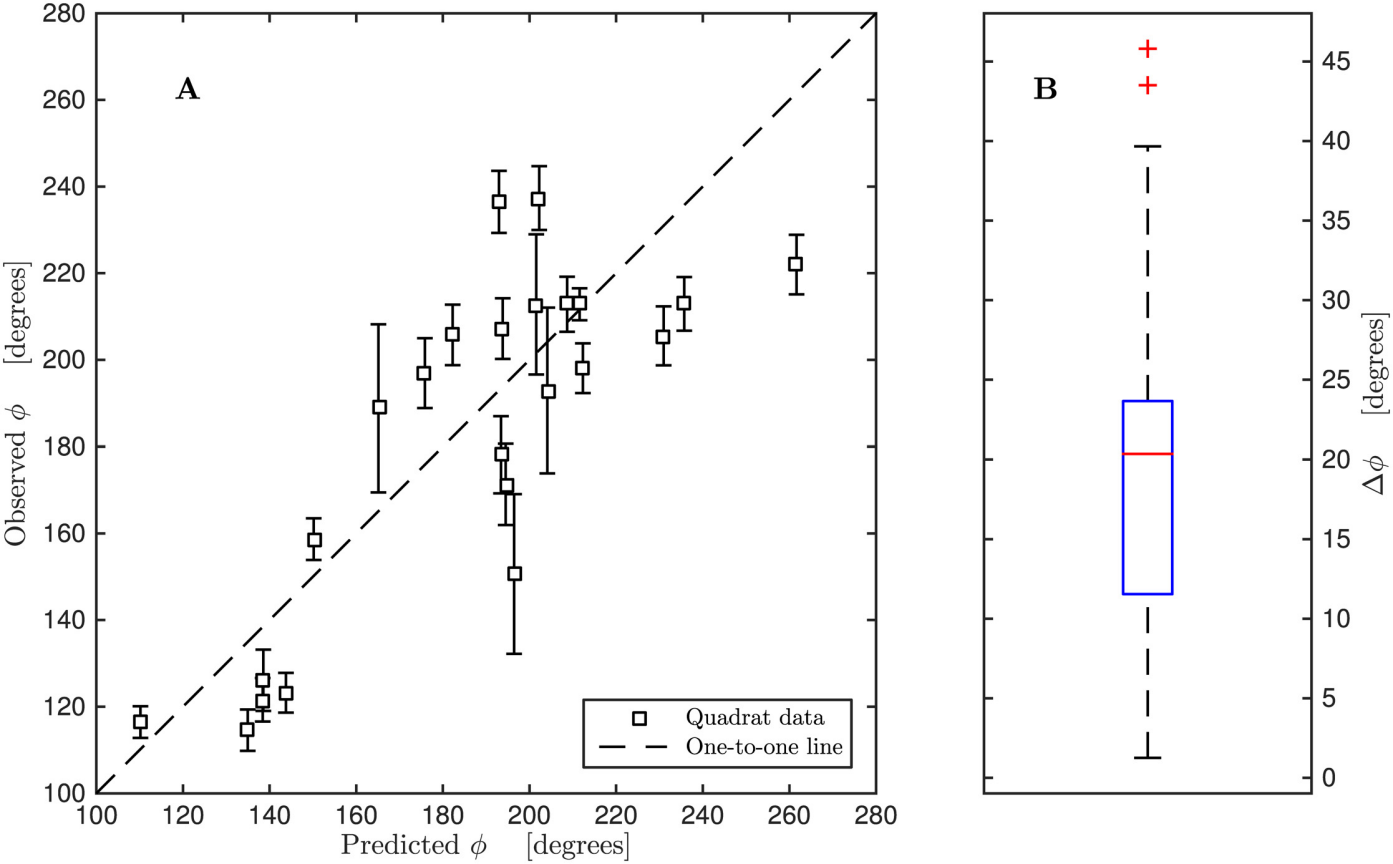
Quantitative assessments of model agreement with observed orientations. (A) Predicted *vs*. observed microtrichial orientation. Data points are scattered narrowly about a one-to-one line, exhibiting a coefficient of determination *R* ^2^ = 0.64 and *p* = 0.001. *ϕ* is microtrichia orientation as defined in Fig 2. Error bars are twice the standard error of the mean orientation in each quadrat. (B) The median absolute difference between modelled and observed microtrichial orientation is 20°. The model predictions are never more than 46° different from observed values, and 75% of the model predictions display residuals less than 25° from observations. The red crosses denote two outlier data points farther than 1.5 times the interquartile range from the upper quartile.

Despite these artifacts, Fig 5 shows that the model captures the general trends of measured microtrichial orientation. Model fidelity was also assessed quantitatively. In Fig 7A the predicted microtrichial orientation is plotted against actual microtrichial orientation for the 23 quadrats. The data is randomly and narrowly scattered about a one-to-one line (dotted line), which corresponds to a perfect fit between measurement and prediction, thus confirming that the model shows small bias error. The model displays a coefficient of determination *R* ^2^ = 0.64 against the observed orientation (*p* = 0.001), indicating that the model explains 64% of the observed variance in local orientation.

Fig 7B shows a boxplot of the absolute residuals between predicted and observed angles to the torsional axis. Model predictions are never more than 46° different from observed values, and the median residual is only 20°. Moreover, 75% of model predictions exhibit residuals less than 25°, a further indication of the good agreement with observation.

The similarity between model and observation highlights the explanatory power of our model in relating microstructure orientation to antiwetting function and large-scale wing morphology. However, there is room for improvement. First, we noted a prediction of divergent orientation that was not observed in the anal area (Fig 6B). This was due to the edge-weighting term $\frac{W_{2}}{{\left\|D\right\|}^{2}}D$ being oriented proximally in the anal area and dominating the other terms in Eq (2). The function of edge-oriented microtrichia is to shed water off the wing quickly, but not onto the body. There appears to be a reduction in edge-weighting closer to the wing base; such a feature could be added to the model (*e.g.* by reducing *W*_2_ in the anal area).

Second, we used the direction to the nearest ridge peak as a proxy for the direction of the local topological gradient (upslope direction), but the gradient may actually point in a different direction. Micro Computed Tomography (micro-CT) techniques have been used to create detailed three-dimensional reconstructions of large insect wings [19], however our attempts to employ micro-CT scanning on this smaller species proved unsuccessful. Three-dimensional laser scanning has been used to reconstruct surfaces of insect wings as small as 17 mm long [20] and might be applied to the wings of *P. heteroptera*; such an approach would facilitate construction of a significantly more detailed and complex model, and is beyond the scope of this study. Adding to the difficulty of accurate measurement, the “upslope direction” may also have limited functional consequence in the anal area, as here the wing is highly compliant and the nearest peak is a relatively large distance away (Fig 2E and 2F). The upslope term *W*_1_ could be made dependent on the distance to or between ridge peaks; nevertheless, the constant value for *W*_1_ derived from Fig 4 proved sufficient to capture the variation in highly-corrugated areas (Figs 5 and 6A). Any dependence of *W*_1_ and *W*_2_ on distance to peaks and distance to the wing root requires further investigation to justify its implementation.

Finally, the weighting factors *W*_1_ and *W*_2_ in Eq (2) could only be determined empirically, but we would prefer to estimate each weight based on thermodynamic or geometric principles. Such a model would have predicitive as well as explanatory power, being able to anticipate microstructure organization in a host of insects given other physical and morphological parameters.

Although opportunities for improvements remain, the simple model implemented in the present study was able to capture, both quantitatively and qualitatively, most aspects of local microtrichial orientation in this species. By employing constant rather than variable weighting values, our model possesses appealing simplicity and a reduced number of variables. The ability to predict local microstructural orientation accurately based on higher-order wing morphology and functional arguments supports the role of microtrichia as directional antiwetting devices. This ability also highlights local variation in orientation as a balance between different, and sometimes competing, antiwetting strategies.

## Conclusions

Previous studies have demonstrated that microstructures on insect wings promote directional drop shedding, but exhibit complex variation in orientation throughout the wing. We have proposed a simple two-dimensional mathematical model to explain local variation in microstructure orientation as a balance between differing adaptive pressures in terms of antiwetting: (1) keeping water raised above the surface and preventing pinning in grooves (*upslope orientation*), (2) shedding water off the wing as quickly as possible (*nearest-edge orientation*), and (3) shedding water away from the body (*distal orientation*). We determined the relative importance of these adaptive pressures in the dipteran *Penthetria heteroptera* through optimization of weighting factors compared to observed local orientation sampled on the dorsal side of the wing. The model explained the majority of the variation of orientation observed when the optimal weights were applied, which supports the role played by microtrichia as directional antiwetting devices. The simple model that we have presented can be readily translated to other insects with directional microstructures, and underscores the importance of considering both function and local wing geometry to explain the organization of natural microstructure arrays.

## Supporting Information

S1 Fig A high-resolution light-microscope compound image of a *P. heteroptera* male left wing. The wing margin and ridge peaks were digitized according to the pixel coordinates in S1 File. Microtrichia orientation was measured on this compound image using quadrat sampling, centred at the pixel coordinates given in S2 File.
(PNG)

S1 File This file provides pixel coordinates (in order) used to digitize the wing margin and ridge peaks. For the case of the ridge peaks, each ridge starts at a starting node (denoted by “1” in the starting node column) and ends at the coordinate immediately prior to the next starting node. Pixel coordinates refer to S1 Fig. The first pixel column on the left of the image is X = 0 and X increases towards the right. The first pixel row on the top of the image is Y = 0 and Y increases towards the bottom.
(CSV)

S2 File This file provides the locations of the centre of each quadrat used for sampling microtrichia orientations. Quadrats are 75 px by 75 px. Pixel coordinates refer to S1 Fig. The first pixel column on the left of the image is X = 0 and X increases towards the right. The first pixel row on the top of the image is Y = 0 and Y increases towards the bottom. Quadrats are numbered as in S3 and S4 Files.
(CSV)

S3 File This file provides the orientations (*ϕ*, in degrees) of each microtrichia sampled in each quadrat. Quadrats are numbered as in S2 and S4 Files. All *ϕ* are standard angles measured from the image positive X axis, according to a cartesian coordinate system where Y is vertical in the image (see Fig 2 in the manuscript).
(CSV)

S4 File This file provides the orientations (*ϕ*, in degrees) at each quadrat location as determined through the model using the optimal values of W1 and W2. Quadrats are numbered as in S2 and S3 Files. All *ϕ* are standard angles measured from the image positive X axis, according to a cartesian coordinate system where Y is vertical in the image (see Fig 2 in the manuscript).
(CSV)
