# Supplementary figures and images for: A Mathematical Model to Capture Complex Microstructure Orientation on Insect Wings
Source: PLoS One. 2015 Oct 7;10(10):e0138282. doi: 10.1371/journal.pone.0138282 (PMC4596840; doi:10.1371/journal.pone.0138282)

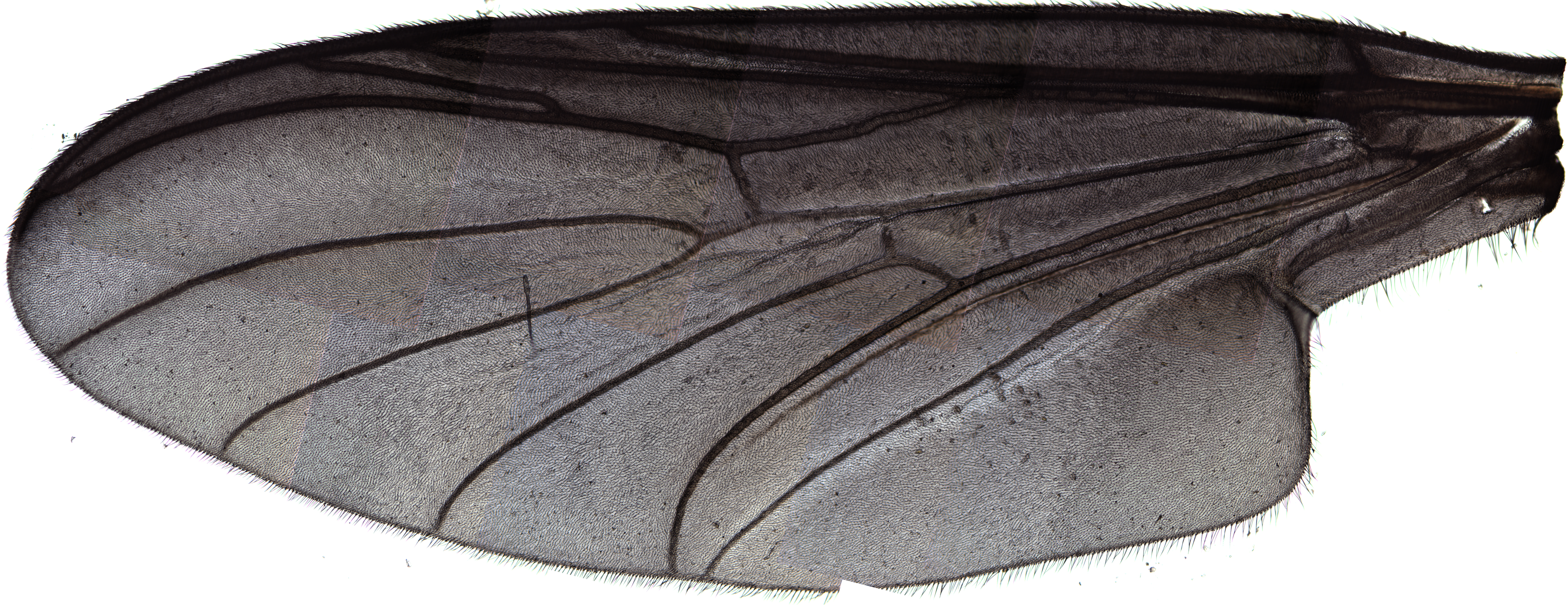

Supplement: S1 Fig — The wing margin and ridge peaks were digitized according to the pixel coordinates in S1 File. Microtrichia orientation was measured on this compound image using quadrat sampling, centred at the pixel coordinates given in S2 File. (PNG) [file pone.0138282.s001.png]
